# Deep-learning survival analysis for patients with calcific aortic valve disease undergoing valve replacement

**DOI:** 10.1038/s41598-024-61685-0

**Published:** 2024-05-13

**Authors:** Parvin Mohammadyari, Francesco Vieceli Dalla Sega, Francesca Fortini, Giada Minghini, Paola Rizzo, Paolo Cimaglia, Elisa Mikus, Elena Tremoli, Gianluca Campo, Enrico Calore, Sebastiano Fabio Schifano, Cristian Zambelli

**Affiliations:** 1 Istituto Nazionale di Fisica Nucleare (INFN), Ferrara, Italy; 2 Maria Cecilia Hospital, GVM Care and Research, Cotignola, Italy; 3 Department of Environmental and Prevention Sciences, Università di Ferrara, Ferrara, Italy; 4 Department of Translational Medicine, Università di Ferrara, Ferrara, Italy; 5 Laboratory for Technologies of Advanced Therapies (LTTA), Ferrara, Italy; 6 Azienda Ospedaliero-Universitaria di Ferrara, Ferrara, Italy; 7 Department of Engineering, Università di Ferrara, Ferrara, Italy

**Keywords:** Data processing, Machine learning, Calcification

## Abstract

Calcification of the aortic valve (CAVDS) is a major cause of aortic stenosis (AS) leading to loss of valve function which requires the substitution by surgical aortic valve replacement (SAVR) or transcatheter aortic valve intervention (TAVI). These procedures are associated with high post-intervention mortality, then the corresponding risk assessment is relevant from a clinical standpoint. This study compares the traditional Cox Proportional Hazard (CPH) against Machine Learning (ML) based methods, such as Deep Learning Survival (DeepSurv) and Random Survival Forest (RSF), to identify variables able to estimate the risk of death one year after the intervention, in patients undergoing either to SAVR or TAVI. We found that with all three approaches the combination of six variables, named albumin, age, BMI, glucose, hypertension, and clonal hemopoiesis of indeterminate potential (CHIP), allows for predicting mortality with a c-index of approximately $80\%$. Importantly, we found that the ML models have a better prediction capability, making them as effective for statistical analysis in medicine as most state-of-the-art approaches, with the additional advantage that they may expose non-linear relationships. This study aims to improve the early identification of patients at higher risk of death, who could then benefit from a more appropriate therapeutic intervention.

## Introduction

Cardiovascular diseases are the leading cause of death in adults^1^. Among them, calcific aortic valve disease (CAVD) leading to a degeneration of valve tissue with a significant impact on hemodynamic changes  ^2,3^, namely aortic stenosis (AS) exhibits an age-dependent increase in prevalence, affecting approximately 5% of the population at 65 years of age^1^.

Aortic valve calcification involves molecular and cellular mechanisms similar to those of atherosclerosis but several clinical studies have shown that the management of those factors, such as dyslipidemia and hypertension, are not effective in slowing the progression of AS delaying the time of intervention^4^. Among genetic factors, Notch1 mutations^5^ or polymorphisms in the LPA (lipoprotein A) gene are involved in the calcification of the aortic valve^3,6,7^ and lipoprotein(a) lowering therapies have been giving promising results in these patients^4^.

As of today, the relevant factors in the progression of CAVD are still mostly unknown^6–9^, there are no standard treatments to slow the progression of valve disease and, thus, AS patients need to undergo surgical aortic valve replacement (SAVR) or transcatheter aortic valve implementation (TAVI)^10^ when the stenosis becomes severe and symptomatic. Nevertheless, following SAVR, life expectancy is lower than in the general population due to an increased relative risk of cardiovascular death^11^ , and one-third to half of patients that underwent TAVI either died or received no symptomatic benefit from the procedure at 1 year  ^12^. In fact, some studies compared the risks related to the SAVR and TAVI for short, intermediate, and long-term follow-up using meta-analysis, and have shown no significant differences in all-cause mortality ^13,14^. Therefore, from our perspective, the patients undergoing either the SAVR or the TAVI procedure both share the same severe cause of disease, namely the AS, and for this reason, to the end of our study, we include both in the same population for life expectancy.

The identification of factors able to predict mortality after valve substitution could be used to develop more tailored treatments to increase survival in patients undergoing SAVR or TAVI or to identify AS patients in which the intervention could be futile. We^15^ and other^16^ have reported that clonal hematopoiesis of indeterminate potential (CHIP), defined as the presence of mutated hematopoietic cell clones in patients without any hematological disease^17^, is linked to an increase of all-cause mortality 1 year after SAVR or TAVI. How the presence of CHIP contributes, together with other factors, to the mortality in AS patients, is currently unknown. Of interest, CHIP is a condition that has been recently linked to a 40% increase in the risk of cardiovascular disease and death, independently from other risk factors^18^.

To gain further insights into specific pathology, medical researchers usually deal with large amounts of complex and intertwined data, including patients clinical and genetic features, interventions, hospitalizations, and follow-ups. In this context, survival analysis is a key activity in investigating the link between individual characteristics or medical procedures and clinical endpoints. Usually, well-known statistics methods such as the Kaplan–Meier (KM) and the Cox Proportional Hazard (CPH), are exploited under the assumption that features are independent and there are multiple linear relationships among them^19^. However, these assumptions are not necessarily true when numerous and complex bio-factors are concerned, and especially when the results depend on a low number of observations^19^.

With the advent of Artificial Intelligence in many scientific fields, recently also Machine Learning (ML) methods came into play to process and find relationships in biomedical data, and to improve survival analysis predictions^20–22^. Different survival ML models such as Deep Learning-based (DeepSurv) and Random Survival Forest (RSF) have been developed and used to evaluate the importance of prognostic variables in predicting patients life expectancy. For example, in^20,21^, authors extensively rely on such methods to predict cancer recurrence after diagnosis or intervention. In particular, Kim et al.^21^ compared the performance of CPH, DeepSurv, and RSF on survival prediction of 255 patients who received surgical treatment for oral cancer. The results of their study suggested that DeepSurv features higher prediction accuracy, allowing this method to guide clinicians in better diagnostic and treatment planning.

One of the main limitations of survival analysis for medical studies concerns data mining since data collection on a large scale is a complex, costly, and time-consuming process^23,24^. Nonetheless, the accuracy of ML methods is tightly bound to the size of the data set and the number of variables involved. In^23^, authors pointed out that in medical research most of the disease modeling and prediction activities address limited-size data, which contrasts the necessity of ML methods to work on large training data. Their approach was based on using multiple model runs and surrogate data analysis. Despite this seems to mitigate the issue, one must bear in mind that the hyper-parameters employed by the ML methods play a substantial role in the performance and reliability of the ML models, and their finding and tuning is a difficult task uncorrelated with the data-set size^25^. Hyper-parameter search methods typically have limited production-strength implementations or do not target scalability on commodity hardware, therefore requesting the use of High-Performance Computing (HPC) platforms^26^.

In this contribution, the focus of our study is on the survival analysis for small-size data sets related to aortic valve calcification. Considering the importance of follow-up for cardiac disease patients and the long necessary follow-up time for CAVD cases, the pivotal goal of such early analysis is to achieve a certain level of how specific biomarkers affect survival probability in a short time. Moreover, investigating whether implementing more advanced statistical methods such as DeepSurv and RSF is capable of providing insights that the traditional methods such as KM and CPH can not.

To reach the goal, we took the benefit of both the conventional statistical analysis and ML approaches and the use of HPC machines. Three different approaches, namely CPH, DeepSurv, and RSF prediction models are exploited, assessing the accuracy of predictions in running them. Also, we discuss how we have tuned the hyper-parameters used in the ML methods we have used that play an important role in the accuracy predictions of the models.

**Table 1:** Statistical characteristics of the full data-set.

Features	Total subjects	p-value
AVR type (TAVI)	54 (33%)	0.262
Sex (male)	80 (48%)	0.147
Smoking	45 (27%)	0.200
Hypertension	136 (73%)	0.154
ASCVD	33 (20%)	0.958
Atrial fibrillation	42 (25%)	0.96
CHIP	49 (30%)	**0.025**
Age	79.15 ± 5.19	0.054
BMI	26.989 ± 3.853	0.093
Hemoglobin	12.832 (11.800–13.800)	**0.048**
Glucose	111.62 (92.000–119.000)	0.260
eGFR	61.58 (46.000–74.000)	0.302
Albumin	4.03 (3.800–4.300)	**0.002**
LDL	89.69 (71.800–103.200)	0.202
LV-EF (%)	59.20 (54.233–66.670)	0.189
Mean aortic gradient	45.93 (39.750–51.000)	0.938
Death (event)	22 (13%)	–
Time (days)	88 (373–448)	–

Results are reported as mean ± standard deviation for normally distributed variables and median and inter-quartile range for non-normally distributed. The entity count and percentage are reported for categorical variables, and statistically significant values (p-value < 0.05) are in bold.

## Methods

In this section, we present the characteristics of our data set used for the survival analysis and the motivations that have led to exploring ML methods along with their optimization in terms of feature selection and hyperparameter tuning.

### Dataset characteristics and statistics at a glance

The work here presented is part of a clinical study named CHARADE to evaluate the association between CHIP and CAVD in the elderly. Medical records of patients were collected at Maria Cecilia Hospital of Cotignola (Italy). The population under study consists of 165 patients undergoing valve replacement for calcific severe aortic stenosis in the time frame from March 2018 to March 2020. Of these, 111 patients had cardiac surgery (SAVR) while 54 patients had TAVI. The study was approved by the Ethics Committee of “Romagna” and was conducted according to the Declaration of Helsinki, and all patients gave written informed consent. The study had a non-interventional retrospective design and all data were analyzed anonymously. The data set analyzed is available from the corresponding authors on motivated request. The data set consists of a relatively large amount of clinical parameters retrieved. Survival was assessed at 12 ± 2 months follow-up after valve replacement. The study variables were downsized from the original dataset to reduce the redundancy, dimension, and complexity of the database. The procedure was performed by the clinician who was in charge of data acquisition, yielding a data set featuring 18 independent variables (death event occurrence, follow-up time, and other 16 clinical features). The selected clinical features are: age, sex, body mass index (BMI), AVR treatment type, smoking status, hypertension presence, atrial fibrillation presence, CHIP, hemoglobin, glucose, Crockoft-gault estimated glomerular filtration rate (eGFR), albumin, low density lipoprotein (LDL) cholesterol, left ventricle ejection fraction (LV-EF), mean aortic gradient, and atherosclerotic cardiovascular disease (ASCVD). This latter variable also includes patients with peripheral artery disease (PAD) and with coronary artery disease such as prior MI, prior CABG, atherosclerotic coronary disease, and prior PCI.

The data-set variables were then split into categorical and numerical to perform statistical analysis using the Scipy-1.4.1 library^27^. For categorical variables, the $\chi ^2$-test with Yates correction has been used. This correction for continuity has been necessary since the event population has been found between 40 and 200. The *χ*^2^ distribution to interpret Pearson *χ*^2^ statistic requires the assumption that the discrete probability of observed binomial frequencies can be approximated by the continuous chi-squared distribution. This assumption is not quite correct and introduces some errors calling for the correction proposed in Yates work^28^. Concerning numerical variables, the Shapiro-Wilk test with a 95% confidence level was performed first to assess the normality of their distribution^29^. The null hypothesis of such a statistical test is that the variable under investigation is normally distributed. If the p-value is less than a chosen confidence level (indicated as $\alpha$), then the null hypothesis is rejected and there is evidence that the variable under consideration is not normally distributed. The Student’s *t*-test^30^ has been applied in the case of normally distributed variables and the Kruskal–Wallis test for the non-normally distributed variables^31^. The former test is a statistical hypothesis test used to test whether the difference between the responses of two groups is statistically significant or not. The latter test is a non-parametric method for testing whether samples originate from the same distribution. It is used for comparing two or more independent samples of equal or different sample sizes.

Table 1 reports the statistical characteristics of the variables in the dataset including the p-value resulting from the tests. Categorical variables are reported with the entity count (i.e., the number of patients having that clinical condition). Numerical variables that are normally distributed are reported with their mean and standard deviation values, whereas for the non-normally distributed variables their median and their interquartile range were reported. A preliminary analysis of the data set shows that the categorical variables are mostly unbalanced and that the numerical features are non-normally distributed except for Age and BMI. To explain better the variables unbalancing in the data set, the normalized value of the data distribution of four variables is shown in Supplementary Material S1. The statistical significance of the variables is reported only for CHIP, hemoglobin, and albumin since their p-value is less than 0.05. Despite the Age variable in the data set having a p-value of 0.054, we can assume that it would play a role in the survival analysis, therefore we include it in the count of statistically significant variables.

### Statistical frameworks for survival analysis

The Kaplan–Meier (KM) estimator is frequently used in survival analysis as a non-parametric method to predict the patients’ lifespan after diagnosis or receiving a treatment for a certain amount of time^32^. Such an estimator has been discussed as a primary tool for survival analysis. The KM statistic ${\hat{S}}(t)$ is defined as

1 $$  {\hat{S}}(t)= \prod_{{t_{i}}<t}{\frac{{n_{i}}-d_{i}}{n_{i}} } $$

where $d_{i}$ and $n_{i}$ are the number of death events at time *t* ascribed to a certain disease and the number of subjects at risk just before time *t*, respectively. When multiple populations of subjects or different subsets of the study group are considered, the KM estimate is complemented by the logrank test^32^. Such a tool is used in survival analysis to test the null hypothesis that there is no difference between the populations in the probability of an event (here a death) at any time point. The analysis is based on the times of event occurrence. By considering two groups of patients on which we want to compare their hazard functions, let $1, \ldots , M$ be the event times observed for each group. Let $N_{1,m}$ and $N_{2,m}$ be the number of patients not yet featuring an event (death) or being censored at the start of period *m* in the two groups, respectively. Let $O_{1,m}$ and $O_{2,m}$ be the number of observed events in the two groups at time *m*, respectively. Define $N_m = N_{1,m} + N_{2,m}$ and $O_m = O_{1,m} + O_{2,m}$. The null hypothesis to be tested is that both groups have the same hazard function so that $H_0 : h_1(t) = h_2(t)$. For all $m = 1, \ldots , M$ we can compute the logrank statistics as:

2 $$\begin{aligned} {Z_i = \frac{\sum _{m = 1}^M \left( O_{i,m}-E_{i,m}\right) }{\sqrt{\sum _{m = 1}^M V_{i,m}}}}\end{aligned}$$

where $i = 1,2$, *E*(*i*, *m*) and *V*(*i*, *m*) are the expected value and the variance of the hypergeometric distribution with parameters $N_m$, $N_{i,m}$, and $O_{m}$. A one-sided level $\alpha$ test will reject the null hypothesis when $Z > z_{\alpha }$, given that $z_{\alpha }$ represents the upper $\alpha$-quantile of the standard normal distribution. The logrank test is based on the same assumptions as the KM survival curve, namely, that censoring is unrelated to prognosis, the survival probabilities are the same for subjects recruited early and late in the study, and the events happened at the times specified^33^. We remind that using the logrank test cannot provide an estimate of the size of the difference between the groups or a confidence interval, but is rather used as a pure test of significance. In this work, we performed the KM estimate and the logrank test on our data set by using the Lifelines-0.26.4 library^34^.

Semi-parametric models are an alternative to non-parametric ones in medical studies^20,21,35^. Their prediction capability is based on the reproducibility of the hazard function, which has been defined as a cumulative function that expresses the probability that the death event will occur within a specific amount of time. The Cox Proportional Hazard (CPH) is a standard semi-parametric model to evaluate the effects of prognostic parameters on the hazard function individually (i.e., univariate) or by combining different factors (i.e., multivariate). This model assumes the linear relationship between the factors, also defined as covariates. The proportional hazard is calculated as

3 $$\begin{aligned} h(t| x) = \lambda _{0}(t) exp \left( \sum _{i=1}^n {\lambda _{i} (x_{i}-\vec {x_{i}}) } \right) \end{aligned}$$

where $\lambda _{0}(t)$ is the time-variant baseline hazard function, and the exponential argument is the log-partial hazard which represents a linear expressed risk function. Thus, the number of covariates affects both the baseline and the partial hazard through specific weight factors. In this work, we performed the CPH model training and fitting on our dataset by using the Lifelines-0.26.4 library^34^. The Kaplan–Meier estimator is particularly useful for estimating the survival function over time, providing a non-parametric way to analyze the probability of an event occurring at or before a given time point. This method is used to estimate the survival probability over time in the presence of censored data. Censored data occurs when individuals are lost to follow-up or the event of interest has not yet occurred by the end of the study. The Kaplan–Meier estimator calculates the probability of survival or median survival time at different time points. It is commonly used for analyzing short-term survival data, such as within a few years. Its disadvantage is that its effectiveness decreases with the increase of the censoring over time. On the other hand, the Cox proportional hazards regression model is a flexible tool for survival analysis, and it can be applied to study the impact of covariates on the hazard of an event preferably for long-term survival scenarios, provided that the proportional hazards assumption is met or appropriately addressed. However, we want to report that there are other studies in the literature that devise Cox regression for short-term analysis given that proper statistical assumptions are met. Examples are the works in^36,37^. In our study, we are limited by the fact that we are dealing with elderly patients and this affects the follow-up time rendering it difficult also to discriminate between early and late mortality. However, the goal of this study is not to provide a detailed gold standard for CAVD analysis, but rather to prove the applicability of Machine and Deep Learning methods in this scenario compared to the standard Cox regression model. In the future, we will try to address all the limitations of this study by working on a longer follow-up time for early and late mortality split.

Despite the importance of CPH in survival analysis, the literature recently highlighted the limits of such modeling strategy in fitting complex survival models^20,21,24,38^. To this extent, approaches based on machine learning and deep learning algorithms started to gain momentum. The basic idea is to analyze all of the variables together to reproduce a dynamic interaction between the frequency of the event happening and variables simultaneously^24^. DeepSurv is a non-linear Cox proportional hazards method based on a neural network^20,38,39^ whose implementation is provided in the PySurvival-0.1.2 library. Here the hazard function in a simplified way can be written as

4 $$\begin{aligned} h(t,\vec {x_{i}}) = \lambda _{0}(t) \psi (\vec {x_{i}}) \end{aligned}$$

where $\psi$ establishing a non-linear risk function among the covariates^40^.

Another popular ML method used for data mining and survival analysis in medical scenarios is a decision tree approach called Random Survival Forest (RSF). The survival model developed in^38^ was used in this study, implemented by the PySurvival-0.1.2 python library. The algorithm grows the survival trees after randomly splitting the original database into the same size samples, setting aside the out-of-bag samples. The average cumulative hazard function (CHF) of all decision trees is used to calculate the overall CHF while the prediction error is calculated only using the out-of-bag samples^40^. The difference between the out-of-bag error rate calculated for the baseline and the permuted model’s performance is defined as variable importance (VIMP). The VIMP has to be mentioned as an important advantage of RSF over other survival models since it provides scalar quantities to measure the variable influence on the model’s prediction accuracy and ranking^41,42^.

### Feature selection

In advance of the survival analysis performed with different methods, variable or feature ranking must be performed to select the optimal number of covariates in the models. In this regard, in this work, we have employed three different techniques based on the assessment of Pearson’s correlation coefficient: the principal component analysis (PCA), and the logrank test associated with a univariate CPH preliminary analysis.

Pearson’s correlation coefficient (indicated as $\rho$) is used to find the redundant variables in the data set by understanding potential linear relationships between them^22^. In this study, we have used the PySurvival-0.1.2 library^40^ with the function for correlation matrix calculation. We consider a suspect or strong linear correlation between features if $\rho \ge |0.5|$, and in this case, the single variables are removed accordingly from the data set.

PCA is then devised to search for strong patterns or data clusters^43^. The goal of the algorithm is to allocate a loading score to the features that contribute to each principal component (PC) and that possibly explains most of the variance in the dataset^44,45^. More specifically, the principal components of a collection of points in a real coordinate space are a sequence of *p* unit vectors, where the $i-th$ vector is the direction of a line that best fits the data while being orthogonal to the first $i-1$ vectors. Many studies use the first two principal components to plot the data in two dimensions and to visually identify clusters of closely related data points^46^. The PCA algorithms of the decomposition module in the Scikit-learn-1.0.1^47^ library were used. The loading scores were calculated for the most important PCs (i.e., PC1 to PC4).

In state-of-the-art survival analysis, the feature selection process also includes the Kaplan–Meier approach, the logrank test, and the assessment of the results provided by univariate and multivariate CPH methods. To evaluate the effects of the 16 features individually on the event of early death over the follow-up time, the univariate CPH linear regression analysis and logrank test were performed with the Lifelines-0.26.4 library^34^. Here, the single variables with a p-value less than 0.05 will be considered as an effective feature for survival prediction. The multivariate CPH was then performed with the same library by adding one covariate at a time to death and follow-up time by which the baseline hazard is calculated. Therefore, to look for the effects of different grouped features, all possible combinations were tested and only those passing the logrank test (p-value < 0.05) were saved. A total number of 214420 feature combinations were found. The results were sorted according to the concordance index (Harrell’s c-index^48^). The best results of each combination with the same feature number were chosen and labeled as a candidate for ML methods application and further optimization. The results of this process are reported in the Supplementary Material S1.

**Figure 1:**
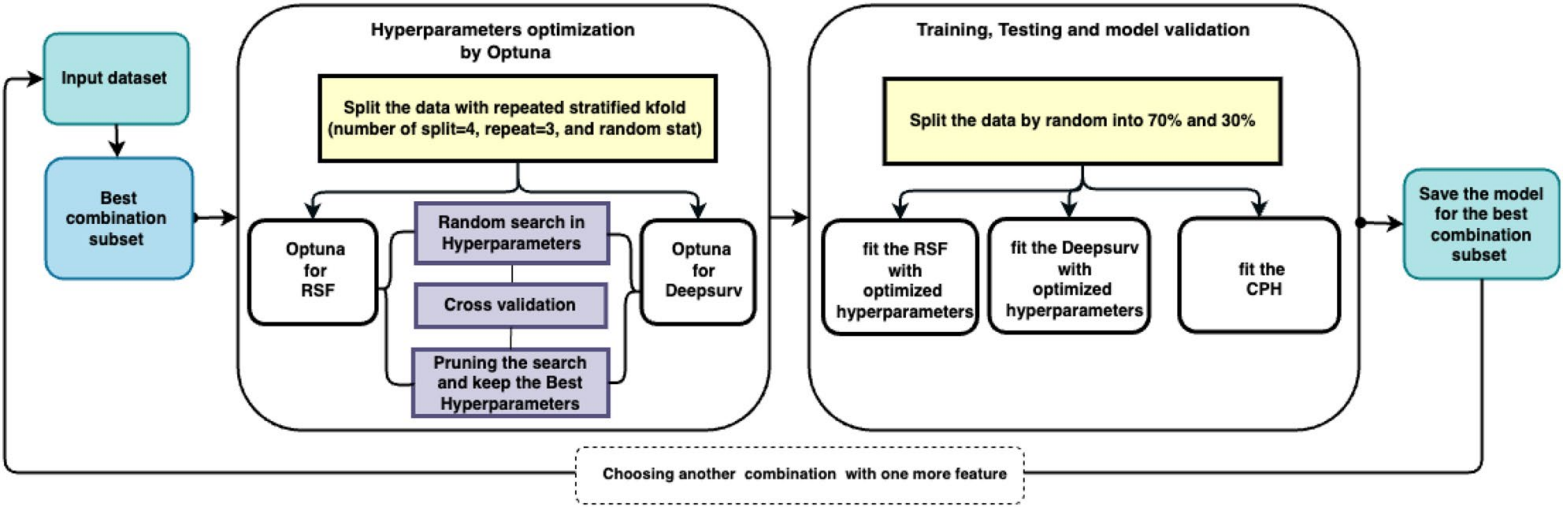
Workflow of our analysis to select the best combination of features, optimize hyperparameters, and perform training of machine learning models. Starting from a set of the three features with the highest c-index, we increasingly add one feature at a time, optimize the hyperparameters with Optuna, train the model, and make a test validation. The train set and test set do not overlap, and repeated stratified k-folding with four splits is used to avoid overfitting of the models.

**Table 2:** DeepSurv and RSF hyperparameters search space value ranges used by the Optuna library to find the best combination that maximizes the test c-index value for both models.

Hyperparameter	DeepSurv values	Hyperparameter	RSF values
Activation	Sigmoid, ReLU, SeLU	Number of trees	100–1000; steps = 100
Layers	1, 2, 3, 4	Max features	sqrt, log_2_
Units	8, 16, 32	Max depth	3–10; steps = 1
Init method	Glorot, uniform	Sample size percentage	0.60–0.85; step = 0.05
Optimizer	Adam, SGD	Importance mode	Impurity corrected, permutation,
Learning rate	10^-5^, 10^-1^		Normalized permutation
L2 reg	10^-6^, 10^-4^		
Dropout	0.1, 0.2, 0.4		

For DeepSurv *Batch Normalization* is fixed to True, *Batch and Dropout* to False, and the *Number of Epochs to 1500*; for RSF the *Min node size* if fixed to 10, and the *Number of Epochs* to 1000.

### Hyperparameters tuning for ML methods

The ML-based models require a training procedure and a subsequent validation process. Both steps rely on optimal hyperparameter settings to increase the c-index and enhance the survival prediction accuracy. An additional challenge is to avoid over- and under-fitting risks typical of ML models dealing with small datasets like that we are using in this work.

Figure 1 shows the steps performed for finding the best hyperparameters, training the models, and searching for the best combination of features giving the highest c-index. The search starts with a set including the 3 features with the highest c-index and increasingly adds one feature at a time and retrains the models. The best combinations are listed in the Supplementary Material S1. To find the best values for the hyper-parameters of the ML models we have used the Python library package Optuna^49^ (version 2.7.0) for each feature combination. Optuna allows for an automatic search of the hyperparameters values space trying to find the best combination that optimizes a user-defined objective function, applying several search strategies, and pruning those combinations that do not improve the objective function, avoiding in this way making exhaustive searches. Despite this, the search process still results very expensively in terms of computing time, and for this reason, we have run the Optuna step on the COKA cluster installed at the University of Ferrara (Italy), a set of High-Performance Computing (HPC) nodes commonly used for scientific numerical simulations. COKA includes several nodes, each equipped with 2 16-core processors, 256 GB of DDR memory, and 16 NVIDIA K80 GPUs.

To account for the issues related to the use of a small dataset, we have applied for a *Repeated Stratified K-fold* strategy. The dataset was split randomly into 75% and 25% with 3 times repetitions as cross-validation, with different randomization as the best trade-off between model accuracy and running time. The Stratified K-Fold is an extension of the regular K-Fold cross-validation where rather than making train and test sets completely random, the ratio between classes in the full dataset is preserved (see Fig. 1 The block of Hyperparameters optimization by Optuna).

The survival predicting models of DeepSurv and RSF were built on the training and test datasets, separately with optimized hyperparameters selection found using the Optuna framework. To overcome the unbalancing in data distribution and minimize the possible bias due to the split of the number of events (death happened in 22 patients, 13%) in the validation dataset, first the entire dataset was divided into two splits of dead (event = 1) and alive (event = 0), then the alive population was split randomly into 70% and 30% splits. The same process was performed for the dead population. By that, the entire dataset is split into four subsets of two trains and two test sets. At last, the training and test sets were concatenated, separately. Therefore, there were no statistical differences between the training and test sets and we are sure the data variance after the split was maintained. In other words, three out of four are used for training, and one for validation, and within each fold the ratio between dead and alive patients is kept equal to that inside the full dataset (see Fig. 1 The block of Training, Testing and model validation).

For each model we have run Optuna with 5000 trials, to search for the best combination values that maximize the c-index of the test set, and for which the maximum brier score (MBS) results in less than the threshold of 0.25 to ensure good accuracy of results^40^. Table 2 lists the value range of each hyperparameter for all models given as input to Optuna.

**Figure 2:**
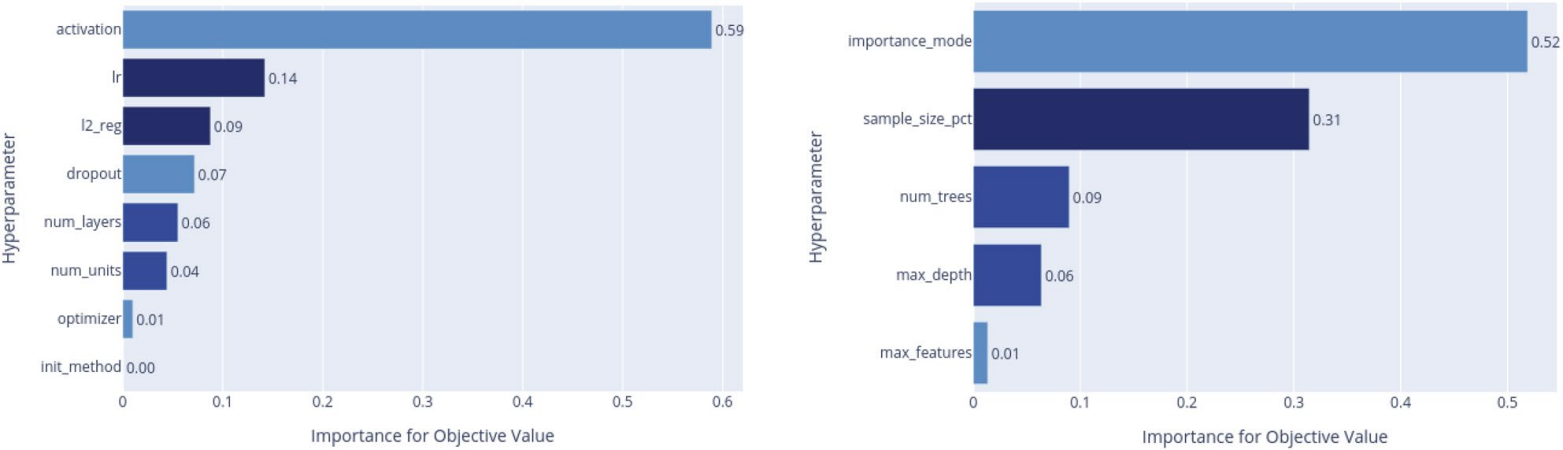
Hyperparameters importance for objective function for DeepSurv (Left) and RSF (Right).

In Fig. 2 we show the hyperparameter importance plots for the objective function resulting from our search process. For the DeepSurv the most relevant are the *activation function* and the *learning-rate* (lr), while for the RSF the *importance mode* and the *sample size percentage* are those with the bigger impact.

**Figure 3:**
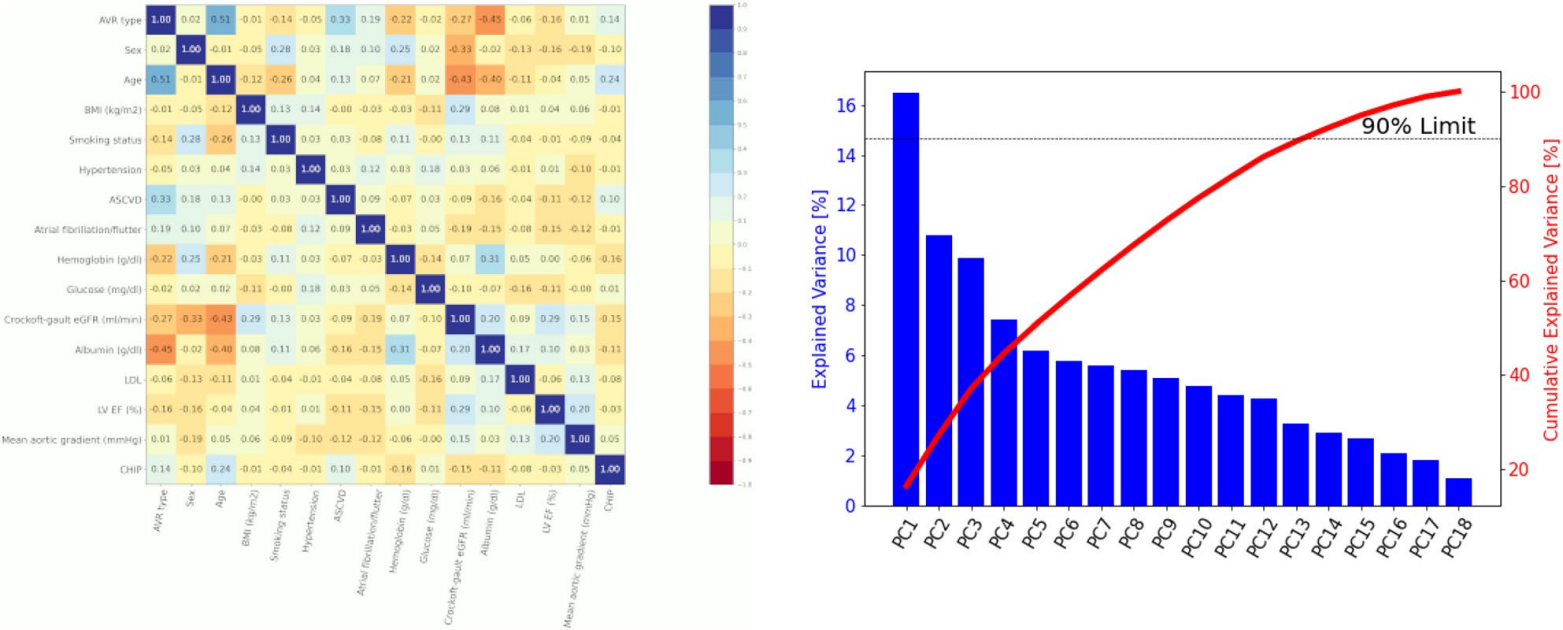
(Left) Pearson’s correlation coefficient as a heat map where the color corresponds to the correlation index. (Right) Scree plot of the PCA with the percentage of variance explained individually and the cumulative value.

### ML models limits and biases

All the ML models considered in this work are subject to limitations and biases^50^ that are disclosed in this section. In this work, we tried to address all the principal limitations although we have to deal with a limited number of patients that represent a *de-facto* bias for the analysis. Once again, we want to highlight that the goal of this study is not to provide a gold standard for the CAVD survival analysis but rather prove, being aware of all the possible biases, that Machine and Deep Learning models can be applied together with the traditional Cox regression models for a superior prediction capability under proper assumptions.

The RSF combines the concepts of random forests and survival analysis techniques. While RSF has become popular due to its ability to handle high-dimensional data like in the case of our dataset, it is important to understand its limitations and biases.

- Censoring Bias is one of the major limitations of RSF since survival analysis involves handling censored data, where the survival time is unknown for some individuals at the end of the study. RSF may underestimate the survival probabilities for censored cases, leading to biased estimations^38^.
- Variable Importance bias is also taken into account for RSF when the most influential predictors are to be determined. Indeed, these important measures can be biased in certain scenarios. For instance, RSF tends to assign more importance to predictors with more unique split points, which may not always reflect their true importance in survival analysis^51^.
- Random forests, including the RSF algorithm, are known for their black-box nature, making it difficult to interpret the underlying decision-making process. RSF can predict survival outcomes accurately; however, understanding the specific relationship between predictors and survival times may be challenging^52^.
- RSF assumes that the observations are independent and identically distributed, which may not always hold in real-world survival analysis scenarios. If the population under study exhibits heterogeneity, RSF may not capture the underlying dynamics accurately, leading to biased estimations^53^.

While DeepSurv has shown promising results in various domains even more than RSF, it also has some limitations and biases that need to be considered as well.

- DeepSurv assumes that censoring is non-informative, meaning that the probability of censoring is independent of the survival time. However, in practice, censoring can be dependent on unobserved characteristics related to the event, introducing bias into the model predictions. This bias can impact the accuracy of survival predictions^20^.
- DeepSurv requires a considerable amount of data to train accurate survival models. Insufficient data can lead to overfitting or poor generalization in predictions. Additionally, the availability of large-scale labeled survival data for training deep learning models is limited, making it challenging to use DeepSurv in specific domains where data is scarce^20^.
- Since RSF and DeepSurv are both black-box models, they share the lack of interpretability issue^54^.
- DeepSurv’s performance is highly affected by the quality and representativeness of the training data. If the training data suffers from sampling bias, the model’s predictions may be biased and not generalize well to unseen data^20^. We addressed this specific issue in this work by using proper training procedures.

## Results

In this section, we report the analysis performed on the full dataset, starting from the knowledge of the features included in the survival analysis models up to a comparison between ML methods and state-of-the-art CPH.

### Covariates insight

In Fig. 3 we show on the left the Pearson correlation plot, and on the right the results of the PCA analysis. The correlation plot does not show strong correlations and linear dependencies between features. The $\rho$ value for *Age* and *AVR type* is notable (0.51), which is ascribed to the *TAVI * procedure preference in elders. However, the correlation is not sufficiently high to claim a marked statistical relationship. Other notable correlations found in the dataset are between *Albumin* and *TAVI* (0.45), and between *Crockoft-gault eGFR* and *Age* (0.43). Since also in this case there is no strong correlations, the dataset is ready to be used by survival analysis algorithms. Then, we performed a PCA analysis to understand data variance and how much a principal component contributes to the explanation of it. As the *Scree plot* evidence, the PC1 only explains about 16.5% of the variance. To explain at least 50% of the data variance, 5 PCs are necessary, whereas to increase the cumulative variance explanation up to 90% at least 13 PCs are needed. Further, we investigated which variables are significant on the relevant PCs. The results shown in Table 3 Left do not evidence a specific set of features in our dataset that explains a possible distribution into different clusters.

**Figure 4:**
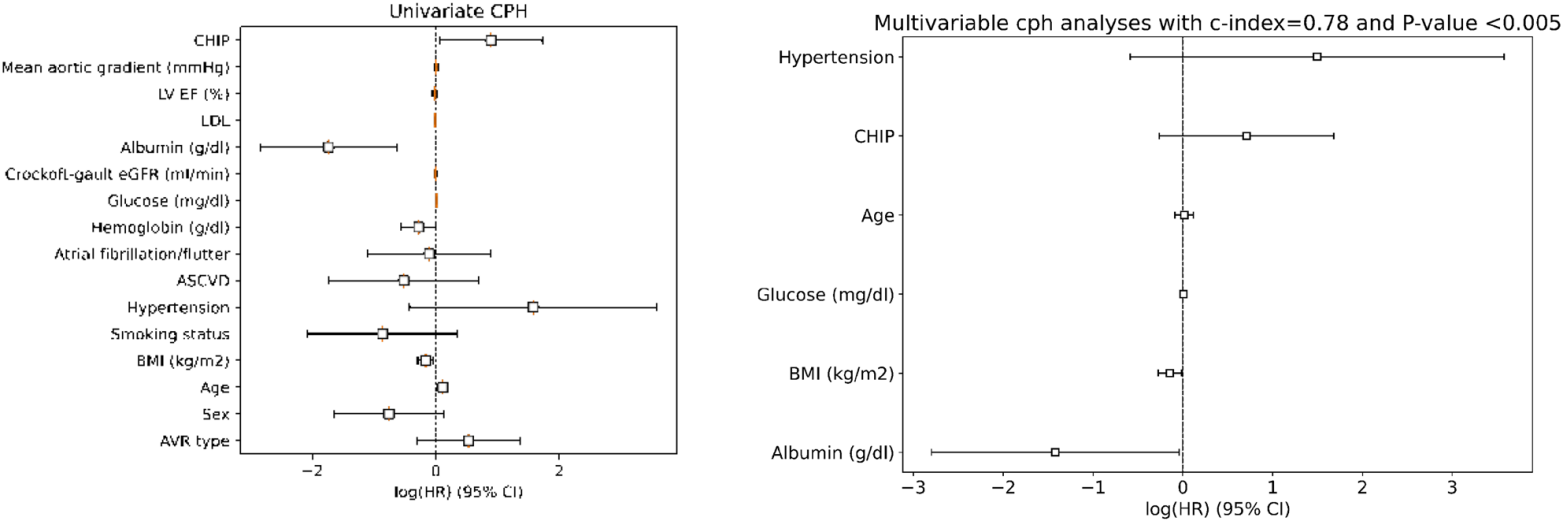
Univariate (left) and multivariable (right) CPH analyses. The hazard ratio is reported in logarithmic scale and 95% confidence interval (CI).

**Table 3:** (Left) PCA loading scores, the highest values for each PC are highlighted in bold, (right) univariate CPH results for each clinical variable.

Features	PC1	PC2	PC3	PC4
Age	**0.397**	0.050	0.251	0.024
Albumin	**0.382**	0.018	0.115	0.131
AVR type	0.377	0.161	0.288	0.111
Crockoft-gault eGFR	0.347	0.241	0.115	0.313
Hemoglobin	0.223	0.154	0.222	0.264
LV EF(%)	0.195	0.296	0.226	0.149
BMI	0.191	0.024	0.194	0.503
ASCVD	0.180	0.272	0.096	0.290
Smoking	0.175	0.147	0.286	0.218
Atrial fibrillation	0.169	0.182	0.102	0.197
CHIP	0.161	0.070	0.165	0.008
Glucose	0.151	0.159	0.288	0.171
LDL	0.141	0.010	0.181	0.287
Mean aortic gradient	0.068	0.241	0.297	0.136
Sex	0.053	**0.427**	**0.344**	0.039
Hypertension	0.016	0.083	0.244	**0.469**

Features	p-value	c-index	logrank test	
AVR type	0.221	0.54	0.21	
Sex	0.085	0.60	0.09	
Age	**0.010**	0.65	–	
BMI	**0.008**	0.70	–	
Smoking status	0.124	0.59	0.15	
Hypertension	**0.047**	0.57	0.09	
Atherosclerotic cardiovascular disease	0.374	0.54	0.40	
Atrial fibrillation/flutter	0.832	0.51	0.83	
Hemoglobin	0.051	0.61	–	
Glucose	**0.017**	0.57	–	
Crockoft-gault eGFR	0.470	0.57	–	
Albumin	**0.003**	0.71	–	
LDL	0.191	0.59	–	
LV-EF%	0.340	0.55	–	
Mean aortic gradient	0.713	0.47	–	
CHIP	**0.039**	0.59	**0.03**	

The statistically significant values (p-value < 0.05) are in bold.

**Figure 5:**
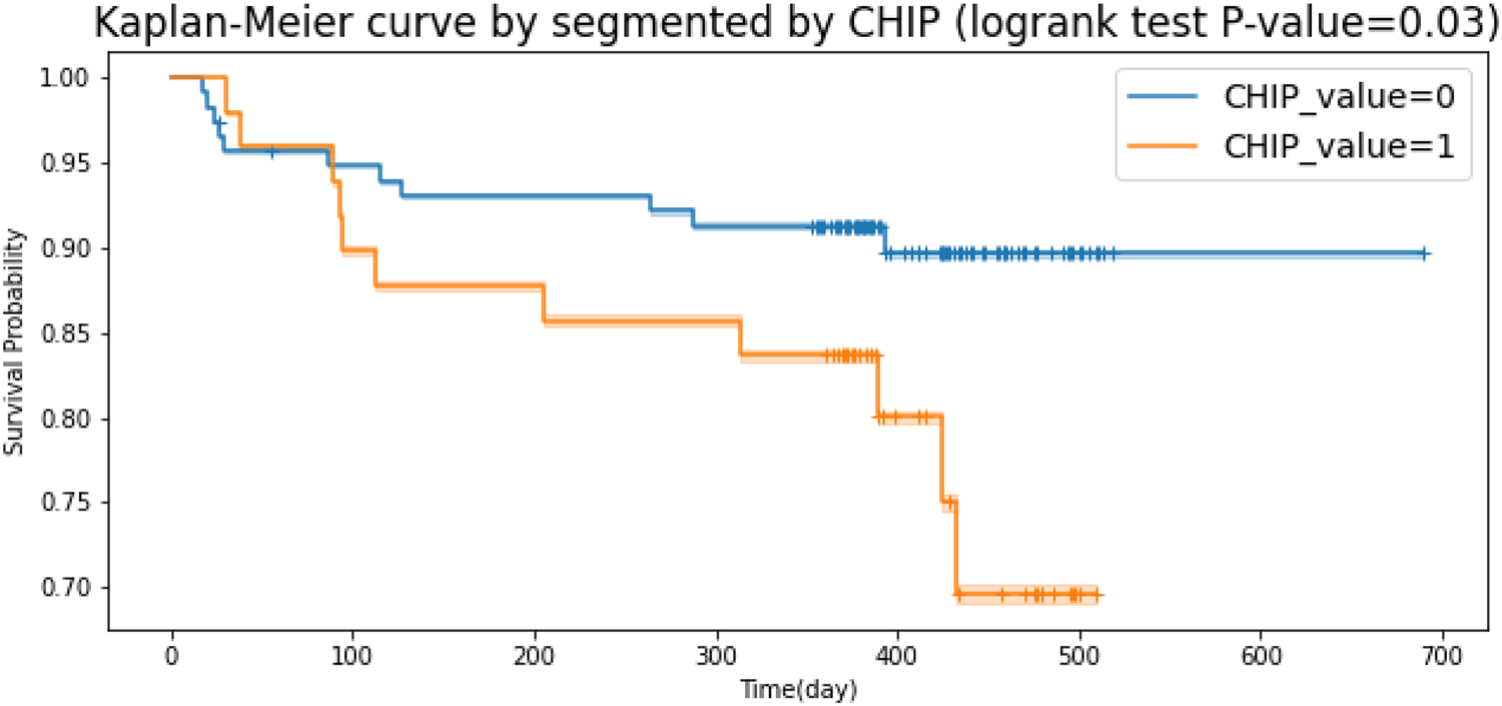
Kaplan–Meier plot segmented by CHIP. The logrank test evidences a p-value of 0.03.

The univariate CPH was then performed for all the covariates in the dataset to understand which of them can be significant in a survival analysis context through the assessment of their hazard ratio. Figure 4 Left shows the logarithm of their hazard ratio value along with the 95% confidence interval. The c-index was computed for each variable and reported in Table 3 Right; as shown, six variables were found significant: *Albumin, Age, BMI, Glucose, CHIP* and *Hypertension*.

**Figure 6:**
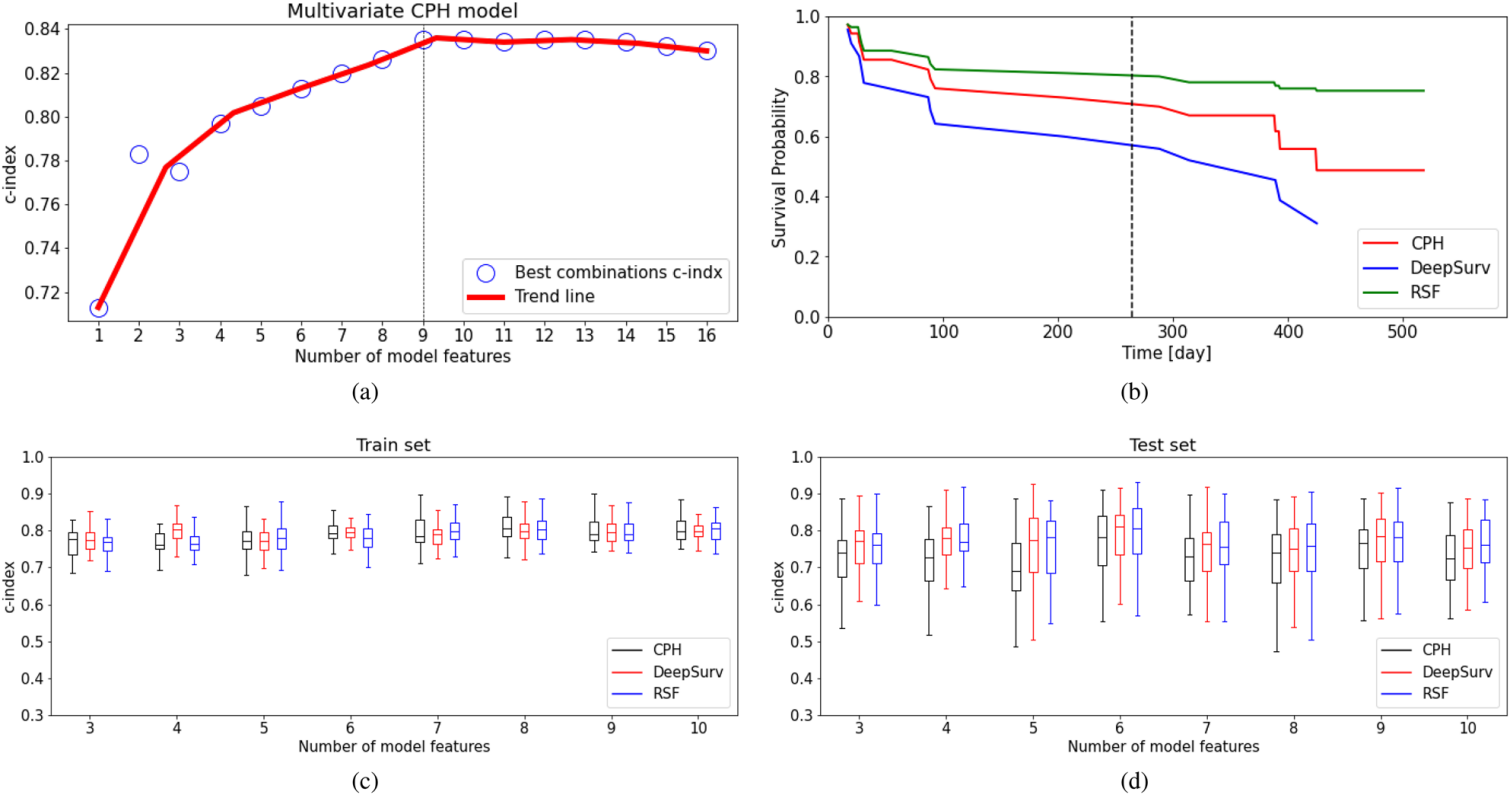
(**a**) The best multivariate CPH c-index for each number of combinations trained with the entire dataset. (**b**) Comparison of the performance of DeepSurv, RSF, and CPH model in terms of survival probability calculated for an example patient. The dashed line represents the actual event time. (**c**) Comparing the statistics of the c-index for the train set as a function of the number of model features evidencing the median, first and third quartiles, and upper and lower bounds. (**d**) Same as the previous case but considering the test set.

All these variables were also tested in multivariate CPH analysis. The c-index for this covariates combination is equal to 0.78. Subsequently, we performed a logrank test specifically for the categorical covariates to understand which have the potential to describe the population subgroups (i.e., survival probability for censored or dead patients). Here, only *CHIP* was found significant through a logrank test (p-value < 0.05). KM survival curves were then plotted accordingly, as shown in Fig. 5. However, according to the table in the Supplementary material S1, there is another combination of six covariates which maximizes the c-index up to 0.8. By using the best combination search strategy described in the previous section of this work, we found the statistically significant multivariate CPH with the maximum possible c-index for an incremental number of features. Figure 6a shows that running a multivariate CPH survival model with more than 9 features does not improve the c-index, since a larger number of statistically insignificant features is added.

### Assessement of ML models performance

To assess the performance of DeepSurv and RSF we have trained both models with the combination of features described in section “Methods”, and compared the prediction accuracy with a multivariate CPH model used as a reference classic model. For all models, the full dataset has been split randomly into two sets, 70% is used for training and 30% for test validation, ensuring that each set contains a similar number of survivors. The multivariate CPH model has been built starting with three statistically significant covariates (i.e., *Glucose, Albumin* and *CHIP*), and then we have increasingly incremented the number up to 16, following an approach similar to that presented in^21^. The same approach has been then also applied to the ML methods.

The c-index achieved as a function of the covariates number by the CPH is shown in Fig. 6a. Results evidence that a combination with 5 covariates is good enough to reach a c-index of approximately 80%. The maximum c-index value of 0.835 is reached with 9 features and then remains constant.

Figure 6b shows a survival probability plot predicted by the three models for a single patient for which the event (death) time occurs at day 264 of the follow-up period. As shown in the figure, the DeepSurv model exhibits higher prediction accuracy compared to the RSF and CPH, since a lower patient survival probability (<0.6 for DeepSurv, $>0.75$ for RSF and CPH) is reported at the event time.

To further assess the prediction accuracy of the ML models, we have run 50 trials per each combination of model features with different random splits of the full dataset, and compared the distribution of the c-index achieved for train and test. Figure 6c shows that for all the models the train c-index slightly improves as the number of features increases, following the trend previously discussed. On the other hand, Fig. 6d proves that the ML models have a superior prediction capability concerning the test set for the data that models have not seen in training, e.g. with 5 features the c-index for CPH is approximately 0.69, while for both ML methods are 0.76.

## Discussion

Aortic valve replacement is recommended for most patients with symptomatic aortic valve disease. Nevertheless, both SAVR and TAVI are associated with relatively high post-procedure mortality, and, thus, the knowledge of predictors for post-AVR survival could be helpful for the identification of novel approaches to improve the management of these patients. Here we report the results of multivariable CHP analysis showing that the combination of six variables (albumin, age, BMI, glucose, hypertension, and CHIP) can predict mortality 1 year after aortic valve replacement, with a c-index of 0.78, in a population of 165 patients that underwent SAVR or TAVI.

The difference between the outcomes of TAVI and SAVR in 6891 patients with low and intermediate-risk patients has been studied after short and intermediate-term follow-up ^14^. In another similar purpose study, the risk outcomes for 20224 patients with moderate and high risk have been studied after a short and long-term follow-up ^13^. Both studies reported no significant differences in their follow-up time. According to our findings, in Table 1, the number of patients undergoing TAVI is 54 out of 165 total patients and the p-value is 0.26 (>0.05). In Table 3, the log-rank test is equal to 0.21 and the p-value for univariate CPH is 0.22 which is larger than 0.05 and is not statistically significant in our study. The PCA analysis we have performed in our dataset does not show AVR type as a parameter that divides our patients into different subgroups, and for this reason, we have managed both in the same way.

A large body of evidence supports a link between albumin level, age, BMI, diabetes mellitus (DM), and mortality following aortic valve replacement. In healthy subjects and patients with acute or chronic illness, serum albumin concentration is inversely related to mortality risk^55,56^ and pre-procedural serum albumin level was found to be independently associated with 1-year mortality in patients who underwent TAVI^57–59^. We report, for the first time that albumin can predict mortality also in patients who underwent SAVR. High age has been identified as an independent prognostic factor of 30-day all-cause mortality after discharge from emergency department^60^, intensive care^61^, and of both in-hospital and post-discharge mortality rates in patients with first myocardial infarctions^62^. In 351 patients that underwent SAVR, the death risk at 5 years was 10%, 20%, and 34% in patients aged < 70 years, 70–79 years, and 80 years, respectively^63^.

Being overweight is associated with improved survival following TAVI when compared with normal weight^64^. Underweight SAVR patients (BMI<20) show an increased hazard ratio (1.519; 95% confidence interval 1.028–2.245) with regards to all-cause mortality at the longest follow-up compared with normal weight patients^65^ and underweight in patients who underwent TAVI is associated with increased mortality^66^. Diabetes mellitus (DM) was associated with poor medium- to long-term overall survival after TAVI^67^ and it remained a strong risk factor for reduced 10-year survival after valve surgery^68^. Among patients with initial asymptomatic mild-to-moderate aortic stenosis, hypertension was associated with more abnormal left ventricular structure and increased cardiovascular morbidity and mortality but no impact on aortic valve replacement was found and there is moderate evidence linking hypertension and early mortality in aortic valve replacement^69^. Recently CHIP was found to be associated with increased mortality, after 1 year after valve replacement, in TAVI and SAVR patients^15,16^.

It is worth noting that in the list of variables related to long-term survival, some are identified in our study such as age, gender, and comorbidities like arterial hypertension^69^, and some not like LV EF%^70^. The reason why we did not use LV EF% is due to the lack of significance and correlation between LV EF and death. In this regard, we refer to the Pearson correlation reported in Fig. 3, and the p-values of 0.189 and 0.34 reported in Table 1 (p-value resulting from the t-tests) and Table 3 (p-value resulting from the log-rank test), respectively. Of course, this may be due to biases in our dataset due to a reduced number of patients unrolled, the high average age of the study population (79.15 ± 5.19 years), and the relatively short-term follow-up. However, we would like to underline that the major aim of our work is how machine learning methods can be applied to make survival analysis and compare to classical analysis. Additionally, we found that DeepSurv exhibits higher prediction accuracy compared to RSF and CPH since a lower patient survival probability ($< 0.6$ for DeepSurv, $> 0.75$ for RSF and CPH) is reported at the event time. These data provide further evidence that ML methods are effective as state-of-the-art approaches broadly used for statistics in medicine, with the advantage that they may expose non-linear relationships and improve c-index, as also reported by others^71^.

The findings of this study could help identify more precisely patients at higher risk of death who could benefit from a more appropriate therapeutic intervention for the modification of the above-cited risk factor. For example, under specific conditions, hypertension could be a factor involved in reduced survival after valve substitution. Furthermore, these findings could reveal previously unrecognized interaction between CVD risk factors in influencing the survival of patients after valve replacement, shedding light on the role of CHIP in increasing the risk of cardiovascular disease and death.

Of interest, in our study, only CHIP was found significant through a log-rank test (p-value < 0.05) confirming the importance of this factor in affecting mortality following AVR. Our results, showing that CHIP together with 5 other factors, four of which (old age, low levels of albumin, and DM) are characterized by chronic pro-inflammatory status^72–74^ suggest the notion of an increased risk of mortality in the studied population due to exacerbated inflammatory condition, based on the results of animal studies^75^ showing that CHIP acts by enhancing the inflammatory response.

Currently, patients are not routinely screened for CHIP since the ratio cost/effectiveness is still high, and for this reason, our dataset features a small number of patients yet is highly dimensional (i.e., with a high number of variables) and informative. However, once validated across diverse and larger patient cohorts, the methodology used in this work can help to assess the survival probability and complement the standard clinical workflow of patients undergoing aortic valve substitution. In addition, the use of machine learning methods can enable the finding of non-linear relationships among bio-factors affecting the success of the clinical intervention. For example, in our case, a biomarker signature, including the CHIP, has been found relevant for predicting the survival probability in patients. Also, machine-learning methods can enhance clinical workflows by providing personalized prognostic information, and supporting informed decision-making based on individual patient data, potentially improving treatment strategies and patient outcomes. Integration could involve, for example, real-time risk assessments and tailored clinical practices.

In conclusion, our work shows how machine learning-based methodologies can be applied to support the analysis of bio-medical datasets, and how the more sophisticated statistical techniques like DeepSurv and RSF can offer insights beyond what conventional methods such as Kaplan–Meier and Cox Proportional Hazard are capable of providing. Moreover, it is ready to be used also to analyze datasets with moderate or long-term follow-ups, once available, overcoming the limitations faced in the current study.

### Supplementary Information

Supplementary Information.

## Data Availability

The raw data supporting the conclusions of this article will be made available by the authors, without undue reservation and upon reasonable request. Please contact the corresponding authors.

## References

[1] Timmis A (2022). European society of cardiology: Cardiovascular disease statistics 2021. Eur. Heart J..

[2] Garg V (2005). Mutations in notch1 cause aortic valve disease. Nature.

[3] Thanassoulis, G. *et al.* Post ws; charge extracoronary calcium working group. genetic associations with valvular calcification and aortic stenosis. *N. Engl. J. Med.* **368(6)**, 503–12. 10.1056/NEJMoa1109034(2013). 10.1056/NEJMoa1109034 PMC3766627 23388002

[4] Shah, S. M., Shah, J., Lakey, S. M., Garg, P. & Ripley, D. P. Pathophysiology, emerging techniques for the assessment and novel treatment of aortic stenosis. *Open Heart* **10**. 10.1136/openhrt-2022-002244 (2023). 10.1136/openhrt-2022-002244 PMC10040005 36963766

[5] Aquila G (2019). The notch pathway: A novel therapeutic target for cardiovascular diseases?. Expert Opin. Ther. Targets.

[6] Libby, P. & Ebert, B. Chip (clonal hematopoiesis of indeterminate potential): Potent and newly recognized contributor to cardiovascular risk. *Circulation* **138(7)**, 666–668. 10.1161/CIRCULATIONAHA.118.034392 (2018). 10.1161/CIRCULATIONAHA.118.034392 PMC6277144 30359133

[7] Mathieu P, Boulanger M (2019). Autotaxin and lipoprotein metabolism in calcific aortic valve disease. Front. Cardiovasc. Med..

[8] Vieceli Dalla Sega, F. *et al.* Cox-2 is downregulated in human stenotic aortic valves and its inhibition promotes dystrophic calcification. *Int. J. Mol. Sci.* **21**. 10.3390/ijms21238917 (2020). 10.3390/ijms21238917 PMC7727817 33255450

[9] Vieceli Dalla Sega, F. *et al.* Cardiac calcifications: Phenotypes, mechanisms, clinical and prognostic implications. *Biology (Basel)* **11**. 10.3390/biology11030414 (2022). 10.3390/biology11030414 PMC8945469 35336788

[10] Toff WD (2022). Effect of transcatheter aortic valve implantation vs surgical aortic valve replacement on all-cause mortality in patients with aortic stenosis: A randomized clinical trial. JAMA.

[11] Glaser N, Persson M, Franco-Cereceda A, Sartipy U (2021). Cause of death after surgical aortic valve replacement: Sweden heart observational study. J. Am. Heart Assoc..

[12] Patel, K. P. *et al.* Futility in transcatheter aortic valve implantation: A search for clarity. *Interv. Cardiol.* **17**, e01. 10.15420/icr.2021.15 (2022). 10.15420/icr.2021.15 PMC8790725 35111240

[13] Carnero-Alcázar M (2016). Transcatheter versus surgical aortic valve replacement in moderate and high-risk patients: A meta-analysis. Eur. J. Cardiothorac. Surg..

[14] Garg A (2017). Transcatheter aortic valve replacement versus surgical valve replacement in low-intermediate surgical risk patients: A systematic review and meta-analysis. J. Invasive Cardiol..

[15] Vieceli Dalla Sega, F. *et al.* Transcriptomic profiling of calcified aortic valves in clonal hematopoiesis of indeterminate potential carriers. *Sci. Rep.* **12**, 20400. 10.1038/s41598-022-24130-8 (2022). 10.1038/s41598-022-24130-8 PMC9701688 36437309

[16] Mas-Peiro S (2020). Clonal haematopoiesis in patients with degenerative aortic valve stenosis undergoing transcatheter aortic valve implantation. Eur. Heart J..

[17] Papa, V. *et al.* Translating evidence from clonal hematopoiesis to cardiovascular disease: A systematic review. *J. Clin. Med.* **9**. 10.3390/jcm9082480 (2020). 10.3390/jcm9082480 PMC7465104 32748835

[18] Libby P (2019). Clonal hematopoiesis: Crossroads of aging, cardiovascular disease, and cancer: Jacc review topic of the week. J. Am. Coll. Cardiol..

[19] RF, W. & WR., C. *Statistical methods for the analysis of biomedical data*, chap. 2nd ed (New York: Wiley-Interscience, 2002).

[20] Katzman JL (2018). Deepsurv: Personalized treatment recommender system using a cox proportional hazards deep neural network. BMC Med. Res. Methodol..

[21] Dong, W. K. *et al.* Deep learning-based survival prediction of oral cancer patients. *Sci. Rep.* **9**, 10.1038/s41598-019-43372-7 (2019). 10.1038/s41598-019-43372-7 PMC6502856 31061433

[22] Chang, S., Abdul-Kareem, S., Merican, A. & Zain, R. Oral cancer prognosis based on clinicopathologic and genomic markers using a hybrid of feature selection and machine learning methods. *BMC Bioinf.* **14**, 170. 10.1186/1471-2105-14-170 (2013). 10.1186/1471-2105-14-170 PMC3673908 23725313

[23] Shaikhina T, Khovanova NA (2017). Handling limited datasets with neural networks in medical applications: A small-data approach. Artif. Intell. Med..

[24] Grossi, E. *Artificial Neural Networks and Predictive Medicine: a Revolutionary Paradigm Shift*, chap. 7 (InTech, 2011). 10.1186/1742-4933-7-S1-S3 PMC3024877 21172062

[25] Balaprakash, P., Salim, M., Uram, T. D., Vishwanath, V. & Wild, S. M. Deephyper: Asynchronous hyperparameter search for deep neural networks. In *2018 IEEE 25th International Conference on High Performance Computing (HiPC)*, 42–51. 10.1109/HiPC.2018.00014 (2018).

[26] Padoin, E. L., Oliveira, D. A. d., Velho, P. & Navaux, P. O. Time-to-solution and energy-to-solution: A comparison between arm and xeon. In *2012 Third Workshop on Applications for Multi-Core Architecture*, 48–53, 10.1109/WAMCA.2012.10 (2012).

[27] Virtanen, P. *et al.* SciPy 1.0: Fundamental Algorithms for Scientific Computing in Python. *Nat. Methods* **17**, 261–272. 10.1038/s41592-019-0686-2 (2020). 10.1038/s41592-019-0686-2 PMC7056644 32015543

[28] Yates F (1934). Contingency tables involving small numbers and the $$\chi ^2$$ test. Suppl. J. R. Stat. Soc..

[29] Shapiro SS, Wilk MB (1965). An analysis of variance test for normality (complete samples). Biometrika.

[30] Student. The probable error of a mean. *Biometrika* **6**, 1–25. 10.2307/2331554 (1908).

[31] Kruskal WH, Wallis WA (1952). Use of ranks in one-criterion variance analysis. J. Am. Stat. Assoc..

[32] Goel M, Khanna P, Kishore J (2010). Understanding survival analysis: Kaplan-meier estimate. Int. J. Ayurveda Res..

[33] Bland JM, Altman DG (2004). The logrank test. BMJ.

[34] Davidson-Pilon, C. lifelines: survival analysis in python. *J. Open Source Softw.* **4**, 1317. 10.21105/joss.01317 (2019).

[35] Bray F (2018). Global cancer statistics 2018: Globocan estimates of incidence and mortality worldwide for 36 cancers in 185 countries. CA Cancer J. Clin..

[36] Ji Q, Tang J, Li S, Chen J (2023). Survival and analysis of prognostic factors for severe burn patients with inhalation injury: based on the respiratory SOFA score. BMC Emerg. Med..

[37] Wang Y (2023). A comparison of random survival forest and Cox regression for prediction of mortality in patients with hemorrhagic stroke. BMC Med. Inform. Decis. Mak..

[38] Ishwaran, H., Kogalur, U., Blackstone, E. & M., L. Random survival forests. *Ann. Appl. Stat.* **2**(3), 841–860. 10.1214/08-AOAS169 (2008).

[39] Wang, H. & Li, G. A. Selective review on random survival forests for high dimensional data. *Quant. Biosci.* **36**(2), 85–96. 10.22283/qbs.2017.36.2.85 (2017). 10.22283/qbs.2017.36.2.85 PMC6364686 30740388

[40] Fotso, S. *et al.* PySurvival: Open source package for survival analysis modeling (2019).

[41] Inglis, A., Parnell, A. & Hurley, C. *Visualizing variable importance and variable interaction effects in machine learning models* **2108**, 04310 (2021).

[42] Dazard, J., Ishwaran, H., Mehlotra, R., Weinberg, A. & Zimmerman, P. Ensemble survival tree models to reveal pairwise interactions of variables with time-to-events outcomes in low-dimensional setting. *Stat. Appl. Genet. Mol. Biol.* **17**(1), 841–860. 10.1515/sagmb-2017-0038 (2017). 10.1515/sagmb-2017-0038 PMC5844232 29453930

[43] Jackson J (1991). A user’s guide to principal components.

[44] Westad, F., Hersleth, M., Lea, P. & Martens, H. Variable selection in pca in sensory descriptive and consumer data. *Food Qual. Prefer.* **14**, 463–472. 10.1016/S0950-3293(03)00015-6 (2003). The Sixth Sense - 6th Sensometrics Meeting.

[45] Ju, J., Banfelder, J. & Skrabanek, L. Quantitative understanding in biology; principal component analysis. https://physiology.med.cornell.edu/people/banfelder/qbio/lecture_notes/3.4_Principal_component_analysis.pdf (2019).

[46] Jolliffe IT, Cadima J (2016). Principal component analysis: A review and recent developments. Philos. Trans. R. Soc. A Math. Phys. Eng. Sci..

[47] Pedregosa F (2011). Scikit-learn: Machine learning in Python. J. Mach. Learn. Res..

[48] Schmid M, Wright MN, Ziegler A (2016). On the use of harrell’s c for clinical risk prediction via random survival forests. Expert Syst. Appl..

[49] Akiba, T., Sano, S., Yanase, T., Ohta, T. & Koyama, M. Optuna: A next-generation hyperparameter optimization framework. In *Proceedings of the 25th ACM SIGKDD International Conference on Knowledge Discovery & Data Mining*, 2623–2631. 10.1145/3292500.3330701 (2019).

[50] Huang Y, Li J, Li M, Aparasu RR (2023). Application of machine learning in predicting survival outcomes involving real-world data: A scoping review. BMC Med. Res. Methodol..

[51] Ishwaran H, Kogalur UB (2010). Consistency of random survival forests. Stat. Probab. Lett..

[52] Strobl C, Boulesteix A-L, Zeileis A, Hothorn T (2007). Bias in random forest variable importance measures: Illustrations, sources and a solution. BMC Bioinformatics.

[53] Mbogning C, Broët P (2016). Bagging survival tree procedure for variable selection and prediction in the presence of nonsusceptible patients. BMC Bioinformatics.

[54] Fernández-Delgado M, Cernadas E, Barro S, Amorim D (2014). Do we need hundreds of classifiers to solve real world classification problems?. J. Mach. Learn. Res..

[55] Akirov A, Masri-Iraqi H, Atamna A, Shimon I (2017). Low albumin levels are associated with mortality risk in hospitalized patients. Am. J. Med..

[56] Goldwasser P, Feldman J (1997). Association of serum albumin and mortality risk. J. Clin. Epidemiol..

[57] Koifman E (2015). Impact of pre-procedural serum albumin levels on outcome of patients undergoing transcatheter aortic valve replacement. Am. J. Cardiol..

[58] Liu G (2020). Meta-analysis of the impact of pre-procedural serum albumin on mortality in patients undergoing transcatheter aortic valve replacement. Int. Heart J..

[59] Hebeler KR (2018). Albumin is predictive of 1-year mortality after transcatheter aortic valve replacement. Ann. Thorac. Surg..

[60] Aasbrenn M, Christiansen CF, Esen B, Suetta C, Nielsen FE (2021). Mortality of older acutely admitted medical patients after early discharge from emergency departments: A nationwide cohort study. BMC Geriatr..

[61] Atramont A (2019). Association of age with short-term and long-term mortality among patients discharged from intensive care units in France. JAMA Netw. Open.

[62] Maggioni, A. P. *et al.* Age-related increase in mortality among patients with first myocardial infarctions treated with thrombolysis. the investigators of the gruppo italiano per lo studio della sopravvivenza nell’infarto miocardico (gissi-2). *N. Engl. J. Med.* **329**, 1442–1448. 10.1056/NEJM199311113292002 (1993). 10.1056/NEJM199311113292002 8413454

[63] Hussain AI (2018). Age-dependent morbidity and mortality outcomes after surgical aortic valve replacement. Interact. Cardiovasc. Thorac. Surg..

[64] Abawi M (2017). Effect of body mass index on clinical outcome and all-cause mortality in patients undergoing transcatheter aortic valve implantation. Neth Heart J.

[65] Forgie K (2020). The effects of body mass index on outcomes for patients undergoing surgical aortic valve replacement. BMC Cardiovasc. Disord..

[66] Voigtländer L (2020). Prognostic impact of underweight (body mass index $$<$$20 kg/m. Am. J. Cardiol..

[67] Lv W (2018). Diabetes mellitus is an independent prognostic factor for mid-term and long-term survival following transcatheter aortic valve implantation: a systematic review and meta-analysis. Interact. Cardiovasc. Thorac. Surg..

[68] Halkos ME (2010). The effect of diabetes mellitus on in-hospital and long-term outcomes after heart valve operations. Ann. Thorac. Surg..

[69] Tjang YS, van Hees Y, Körfer R, Grobbee DE, van der Heijden GJ (2007). Predictors of mortality after aortic valve replacement. Eur. J. Cardiothorac. Surg..

[70] Baranowska O (2012). Factors affecting long-term survival after aortic valve replacement. Kardiol. Pol..

[71] Penso, M. *et al.* Predicting long-term mortality in tavi patients using machine learning techniques. *J. Cardiovasc. Dev. Dis.* **8**. 10.3390/jcdd8040044 (2021). 10.3390/jcdd8040044 PMC8072967 33923465

[72] Sanada F (2018). Source of chronic inflammation in aging. Front. Cardiovasc. Med..

[73] Ronit A (2020). Plasma albumin and incident cardiovascular disease: Results from the cgps and an updated meta-analysis. Arterioscler. Thromb. Vasc. Biol..

[74] Tsalamandris, S. *et al.* The role of inflammation in diabetes: Current concepts and future perspectives. *Eur. Cardiol.* **14**, 50–59. 10.15420/ecr.2018.33.1 (2019). 10.15420/ecr.2018.33.1 PMC6523054 31131037

[75] Fuster JJ (2017). Clonal hematopoiesis associated with tet2 deficiency accelerates atherosclerosis development in mice. Science.

