# Supplementary material for: Deep-learning survival analysis for patients with calcific aortic valve disease undergoing valve replacement
Source: Sci Rep. 2024 May 13;14:10902. doi: 10.1038/s41598-024-61685-0 (PMC11091174; doi:10.1038/s41598-024-61685-0)
Supplement: Supplementary file 1 — Supplementary Information. [file 41598_2024_61685_MOESM1_ESM.pdf]

1 Supplementary information

Data distribution for five candidate variables are illustrated in Fig.1. The Gaussian curve was fitted to the numerical variables. Among the numerical variables, Age is the perfectly normally distributed and LV-EF% is an example of a non-Gaussian like distribution. Among the categorical variables, Sex and Atherosclerotic cardiovascular disease are the candidates for a well-balanced (48% male) and unbalanced (20% with disease) variables.

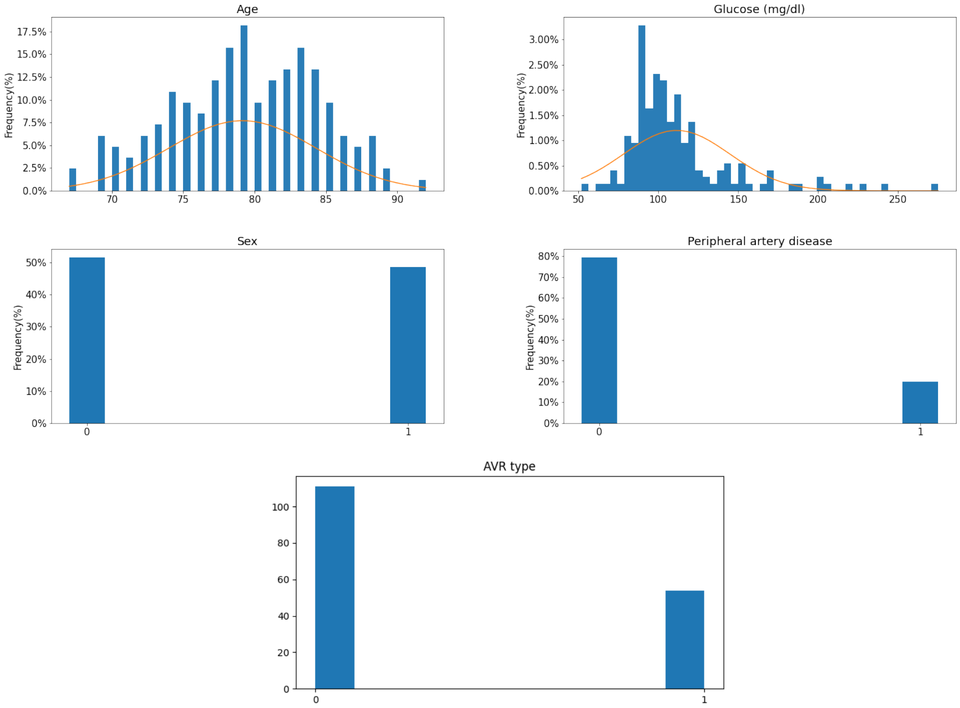

Supplementary Figure 1. Data distribution for five candidate variables representing the entire datasets. The fitted Gaussian curves for the numerical variables were overlapped on the histogram plot in orange.

	Features	Concordance	nEvent	nEntries	Pvalue
1	Albumin	0.713	20	155	0.003
2	Albumin, Glucose	0.783	19	154	0.003
3	Albumin, Glucose, CHIP	0.775	19	154	0.003
4	Albumin, Glucose, CHIP, Smoking status	0.797	19	154	0.005
5	Albumin, Glucose, CHIP, Smoking status, Hemoglobin	0.805	19	154	0.011
6	Albumin, Glucose, CHIP, Smoking status, Hypertension, Atherosclerotic cardiovascular disease	0.813	19	153	0.004
7	Sex, BMI, Glucose, CHIP, LDL, Hypertension, Hemoglobin	0.820	21	159	0.002
8	Sex, BMI, Glucose, CHIP, LDL, Hypertension, Atherosclerotic cardiovascular disease, Hemoglobin	0.826	21	158	0.001
9	Sex, BMI, Glucose, CHIP, LDL, Smoking status, Hypertension, Atherosclerotic cardiovascular disease, Hemoglobin	0.835	21	158	0.002
10	Sex, BMI, Glucose, CHIP, LDL, Smoking status, Hypertension, Atherosclerotic cardiovascular disease, Hemoglobin, Crockoft-gault eGFR	0.835	21	158	0.004
11	Sex, BMI, Glucose, CHIP, LDL, Smoking status, Hypertension, Atherosclerotic cardiovascular disease, Hemoglobin, Crockoft-gault eGFR, Mean aortic gradient	0.834	21	157	0.007
12	BMI, Albumin, Glucose, CHIP, LDL, Smoking status, Hypertension, Atherosclerotic cardiovascular disease, Hemoglobin, Crockoft-gault eGFR, Atrial fibrillation/flutter, AVR type	0.835	19	150	0.009
13	BMI, Albumin, Glucose, CHIP, LDL, Smoking status, Hypertension, Atherosclerotic cardiovascular disease, Hemoglobin, Crockoft-gault eGFR, Atrial fibrillation/flutter, LV EF, Age	0.835	19	149	0.011
14	BMI, Albumin, Glucose, CHIP, LDL, Smoking status, Hypertension, Atherosclerotic cardiovascular disease, Hemoglobin, Crockoft-gault eGFR, Atrial fibrillation/flutter, LV EF, Age, AVR type	0.834	19	149	0.017
15	Sex, BMI, Albumin, Glucose, CHIP, LDL, Smoking status, Hypertension, Atherosclerotic cardiovascular disease, Hemoglobin, Crockoft-gault eGFR, Atrial fibrillation/flutter, LV EF, Age, Mean aortic gradient	0.832	19	148	0.021
16	Sex, BMI, Albumin, Glucose, CHIP, LDL, Smoking status, Hypertension, Atherosclerotic cardiovascular disease, Hemoglobin, Crockoft-gault eGFR, Atrial fibrillation/flutter, LV EF, Age, AVR type, Mean aortic gradient	0.830	19	148	0.030

Supplementary Table 1. The best combinations including 1 to 16 features. nEvent: the number of Observed Events, nEntries: the number of Entries (Non-null rows)
